# Improving Education and Confidence for Junior Doctors Regarding Physical Health Matters on Psychiatry Wards: The Physical Health Huddle

**DOI:** 10.1192/bjo.2022.109

**Published:** 2022-06-20

**Authors:** Cornelia Beyers, Onaiza Awais, Sophie Stokes, Rajesh Moholkar, Alice Packham

**Affiliations:** 1 Birmingham and Solihull Mental Health Foundation Trust, Birmingham, United Kingdom; 2 Guy's and St Thomas' NHS Foundation Trust, London, United Kingdom

## Abstract

**Aims:**

The COVID-19 pandemic highlighted a greater need for multidisciplinary input for psychiatric patients with complex physical comorbidities at Reaside Forensic Medium Secure clinic. It was also felt that junior doctors would benefit from support in managing complex physical health matters as well as issues arising whilst on-call in order to improve morale and support their educational needs. We aimed to add to existing services by offering junior doctors a regular discussion group (Physical Health Huddle) to support with complex cases, share different perspectives on patient treatment and open conversation regarding issues arising whilst on-call. We further hoped to improve communication, provide education for junior trainees with limited experience of forensic psychiatry and support their involvement in patient care and multi-disciplinary meetings.

**Methods:**

Junior doctors were invited to a monthly informal Huddle (in person and online) and supported to propose patients for discussion. A proforma was supplied to assist. The junior doctor presented the summary and following discussion we explored various ideas on how to manage the patient's physical health. Feedback was provided to the patient teams afterwards and short before and after questionnaires were used to monitor effectiveness and collect feedback.

**Results:**

The result showed a significant increase in support felt and individual feedback highlighted the need to continue this effort. The Huddle therefore provided a safe reliable space to freely discuss concerns regarding the day-to day management or escalation of complex physical health issues on psychiatric wards as well as on-call.

**Conclusion:**

The Huddle successfully created a sustainable, effective and interactive short learning session which has shown to be effective in engaging trainees in this vital area and help us meet our aim. This format further has the potential to be refined and rolled out to a wider audience in the future to improve learning throughout the trust regarding physical health matters.

